# Increase of MZB1 in B cells in systemic lupus erythematosus: proteomic analysis of biopsied lymph nodes

**DOI:** 10.1186/s13075-018-1511-5

**Published:** 2018-01-30

**Authors:** Aya Miyagawa-Hayashino, Hajime Yoshifuji, Koji Kitagori, Shinji Ito, Takuma Oku, Yoshitaka Hirayama, Adeeb Salah, Toshiki Nakajima, Kaori Kiso, Norishige Yamada, Hironori Haga, Tatsuaki Tsuruyama

**Affiliations:** 1 Center for Innovation in Immunoregulative Technology and Therapeutics, Graduate School of Medicine, Kyoto University, Yoshida-konoe-cho, Sakyo-ku, Kyoto, 606-8501 Japan; 2 Department of Diagnostic Pathology, Kyoto University Hospital, Kyoto, Japan; 3 Department of Rheumatology and Clinical Immunology, Graduate School of Medicine, Kyoto University, Kyoto, Japan; 4 Bio Frontier Platform, Graduate School of Medicine, Kyoto University, Kyoto, Japan; 5 Research Portfolio & Science, Drug Discovery Research, Astellas Pharma Inc., Tsukuba, Japan; 6 Center for Anatomical, Pathological and Forensic Medical Research, Graduate School of Medicine, Kyoto University, Kyoto, Japan; 7 Present address: Department of Clinical Pathology, Kansai Medical University, Osaka, Japan

**Keywords:** Formalin-fixed paraffin-embedded, Lupus-prone mice, Proteomic analysis, Systemic lupus erythematosus, SLE lymphadenopathy, TUNEL, Unfolded protein response

## Abstract

**Background:**

Systemic lupus erythematosus (SLE) is a prototypical autoimmune disease in which dysregulation of B cells has been recognized. Here, we searched for potential biomarkers of SLE using liquid chromatography-tandem mass spectrometry (LC-MS).

**Methods:**

Lymph nodes from SLE patients and controls were analyzed by LC-MS. To validate the identified molecules, immunoblotting and immunohistochemistry were performed and B cells from SLE patients were analyzed by quantitative RT-PCR. B-cell subsets from NZB/W F1 mice, which exhibit autoimmune disease resembling human SLE, were analyzed by flow cytometry. Endoplasmic reticulum (ER) stress was induced by tunicamycin and the serum concentration of anti-dsDNA antibodies was determined by ELISA. TUNEL methods and immunoblotting were used to assess the effect of tunicamycin.

**Results:**

MZB1, which comprises part of a B-cell-specific ER chaperone complex and is a key player in antibody secretion, was one of the differentially expressed proteins identified by LC-MS and confirmed by immunoblotting. Immunohistochemically, larger numbers of MZB1^+^ cells were located mainly in interfollicular areas and scattered in germinal centers in specimens from SLE patients compared with those from controls. MZB1 colocalized with CD138^+^ plasma cells and IRTA1^+^ marginal zone B cells. *MZB1* mRNA was increased by 2.1-fold in B cells of SLE patients with active disease (SLE Disease Activity Index 2000 ≥ 6) compared with controls. In aged NZB/W F1 mice, splenic marginal zone B cells and plasma cells showed elevated MZB1 levels. Tunicamycin induced apoptosis of MZB1^+^ cells in target organs, resulting in decreased serum anti-dsDNA antibody levels. Additionally, MZB1^+^ cells were increased in synovial tissue specimens from patients with rheumatoid arthritis.

**Conclusions:**

MZB1 may be a potential therapeutic target in excessive antibody-secreting cells in SLE.

**Electronic supplementary material:**

The online version of this article (10.1186/s13075-018-1511-5) contains supplementary material, which is available to authorized users.

## Background

Systemic lupus erythematosus (SLE) is a systemic inflammatory autoimmune disease characterized by production of autoantibodies directed against nucleic acid-associated autoantigens that cause multiple organ damage, including skin, joints, kidney, and the central nervous system [1]. Aberrant innate immune responses play an important role in SLE, contributing tissue injury via release of proinflammatory cytokines and aberrant activation of T and B cells, with the latter leading to pathogenic autoantibody production [2]. Autoreactive B cells differentiate into pathogenic memory and plasma cells via germinal center responses [3]. B-cell depletion is an attractive therapeutic option in SLE. However, targeting human CD20 (rituximab), which is expressed on almost all B-cell lineages except early pro-B cells, plasmablasts, and plasma cells, has shown disappointing results in two randomized clinical trials of lupus [4, 5], although there has been a question about both trial design and clinical outcome measures [2]. Recent clinical trials using belimumab, a fully humanized monoclonal antibody directed against B-lymphocyte stimulator (BLyS; also known as BAFF), proved beneficial [6–9]. Dual B-cell immunotherapy was confirmed to be superior to individual anti-CD20 depletion or BAFF blockade in a murine lupus model [10]. Upon binding to its receptors, TACI, BAFF receptor, and B-cell maturation antigen, BLyS activates signals for B-cell survival and maturation with belimumab effect via depletion of recently formed, rather than memory, B cells or long-lived plasma cells in SLE [6–8]. Recently, proteasome inhibitors bortezomib, delanzomib, and carfilzomib have shown to be effective in refractory SLE patients [11] or lupus-prone mice by depleting plasma cells [12–14]. Plasma cells are sensitive to proteasome inhibitors because of their high rate of antibody synthesis [11–14].

In the study of murine lupus-prone models, the spleen is examined to understand the underlying mechanisms of lupus [15–21]. Although localized or generalized lymphadenopathy is a common symptom, seen at some stage in the evolution of the disease in about 60% of SLE patients [22], for SLE patients lymphoid organs (e.g., spleen and lymph node) are rarely biopsied, only if malignancy should be ruled out. The pathology database in our institution contained five archives of biopsied specimens from patients with lymphadenopathy associated with SLE, which were collected to rule out malignancy. Formalin-fixed paraffin-embedded (FFPE) tissues archived in pathology department laboratories worldwide may provide an extremely valuable resource for biomarker discovery for rare diseases [23]. Recent developments in methodologies using FFPE tissue in mass spectrometry-based proteomics led us to examine potential biomarkers and new therapeutic targets of SLE [24].

Here, we applied liquid chromatography-tandem mass spectrometry (LC-MS) to analyze lymph node tissue from SLE patients and identified MZB1 as a potential biomarker of SLE.

## Methods

### Proteomic sample preparation and LC-MS

SLE lymphadenopathy is lymph node enlargement associated with SLE. Reactive follicular hyperplasia is the most frequent finding [22]. Lymph nodes from patients diagnosed with SLE (*n* = 3) and controls (*n* = 3) were used for LC-MS analysis. The controls were lymph nodes dissected during thyroidectomy for papillary carcinoma with no metastasis found. Proteins from FFPE tissue were extracted using the Liquid Tissue MS Protein Prep Kit (Expression Pathology Inc., Rockville, MD, USA). The extracts were diluted in 0.1% formic acid and 1 μg aliquots for each sample were separated by nanoflow reversed-phase LC (NanoLC-Ultra 2D-Plus; Eksigent, Dublin, CA, USA) equipped with cHiPLC Nanoflex (Eksigent). Eluted peptides were analyzed by a quadrupole time-of-flight hybrid mass spectrometer (Triple TOF5600+ system; AB SCIEX, Framingham, MA, USA).

### Identification and quantification of peptides

Tandem mass spectra were searched against the Uniprot-KB/Swissprot human proteomic database (2014–June) from the European Bioinformatics Institute. ProteinPilot software version 4.5β (AB SCIEX) was used for the database search. False discovery rates (FDRs) were determined after peptide/protein identification using the Proteomic System Performance Evaluation Pipeline provided as a part of ProteinPilot software (AB SCIEX). Label-free quantification of peptides was performed using Progenesis QI for Proteomics software (Nonlinear Dynamics, Newcastle upon Tyne, UK). Protein abundance was determined by the relative quantification using the nonconflicting peptides. Proteins identified by at least two distinct peptides (confidence ≥ 95%) were used in the following analyses. The details of the analysis were described previously [24].

### Validation using immunoblotting in human tissues

Analysis of MZB1, one of the highly confident and differentially expressed proteins between SLE patients and controls in LC-MS, was chosen for a validation study. Immunoblot analysis was performed using proteins extracted from lymph nodes from SLE patients and controls using the Qproteome FFPE Tissue Kit (Qiagen, Venlo, the Netherlands). The antibodies used were MZB1 (Proteintech, Rosemont, IL, USA) and beta-actin (Abcam, Cambridge, UK). Protein bands were visualized using a chemiluminescence substrate (Nacalai Tesque, Kyoto, Japan), and images were obtained using Ez-Capture MG (Daihan Scientific Co., Ltd, Seoul, South Korea). Visualized bands were analyzed using CS Analyzer (Atto Corporation, Tokyo, Japan).

### Patient samples and immunohistochemistry

For immunohistochemistry, the expression profile of MZB1 was investigated by the REAL EnVision/HRP detection system (DakoCytomation, Glostrup, Denmark). Lymph nodes from patients with SLE lymphadenopathy (*n* = 5) or controls (*n* = 5) were used for a validation study of LC-MS results. In addition, excised tonsil specimens from patients with tonsillar hypertrophy (*n* = 4), biopsy specimens for polyarteritis nodosa (skin, *n* = 6; skeletal muscle, *n* = 1), muscle biopsies for dermatomyositis (*n* = 6), lip biopsies for Sjögren’s syndrome (*n* = 7), excisional pancreas biopsies for IgG4-related pancreatitis (*n* = 5), thyroidectomy specimens for Hashimoto’s thyroiditis (*n* = 6), synovial tissues for rheumatoid arthritis (*n* = 10), and renal biopsies for lupus nephritis (*n* = 9, Class IV) were used for comparison with specimens from patients with SLE lymphadenopathy. The samples were taken at Kyoto University Hospital from 2006 to 2014 and stored in FFPE blocks.

The number of MZB1^+^ cells at three different high-power fields (HPFs) was counted in each section, and the average number of positive cells per HPF was calculated. For lymph nodes and tonsils, the count was performed in the germinal center and interfollicular area, respectively.

Double immunofluorescence staining was performed with antibodies against MZB1 and CD20 (Clone L26; DakoCytomation), CD138 (Clone MI15; DakoCytomation), or immunoglobulin superfamily receptor translocation-associated 1 (IRTA1) (FCRL4/FcRH4; R&D Systemes, Minneapolis, MN, USA) and detected using the Opal 2-Plex Kit, Cyanine 5/Fluorescein (PerkinElmer, Inc., Waltham, MA, USA). Nuclei were visualized with DAPI (Dojindo, Kumamoto, Japan). Fluorescence imaging analysis was performed using a fluorescence microscope (FSX100; Olympus, Tokyo, Japan).

### Isolation of human peripheral blood B cells

Peripheral blood was obtained from consenting SLE patients (*n* = 13) and healthy donors (*n* = 6). Patients were assessed using the SLE Disease Activity Index 2000 (SLEDAI-2 K) [25]. Five patients had SLEDAI-2 K ≥ 6 (high activity) and eight patients had SLEDAI-2 K < 6 (low activity). Two patients with high activity had follow-up samples collected at 2 months of treatment. Human peripheral blood mononuclear cells (PBMCs) were isolated from the blood using Lymphocyte Separation Solution (Nacalai Tesque). CD19^+^ B cells were isolated from PBMCs using the MACS Pan B Cell Isolation Kit (Miltenyi Biotec, Bergisch Gladbach, Germany).

### Quantitative real-time PCR in human B cells

cDNA was synthesized using the SuperScript III First-Strand cDNA Synthesis System for RT-PCR (Life Technologies, CA, USA). Quantitative real-time PCR (qRT-PCR) was performed in 384-well plates with TaqMan gene probes and primers designed by Life Technologies for *MZB1* (assay ID: Hs00414907_ml) and *beta-actin* (assay ID: Hs01060665_gl). These reactions were performed using the ViiA 7 Real-Time PCR System (Applied Biosystems, ThermoFisher, Tokyo, Japan) with TaqMan Fast Advanced Master Mix (Life Technologies). *MZB1* mRNA expression was normalized to that of *beta-actin* using the 2^–∆∆Ct^ method.

### Mice

Female [NZB × NZW] F1 (BWF1) and C57BL/6 N (B6) mice were purchased from Japan SLC (Shizuoka, Japan) and maintained in the Kyoto University animal facility. Young mice (10–12 weeks of age) and aged mice (30–34 weeks of age) were used for the study. For tunicamycin (TM) treatment, mice aged 25–30 weeks were used because mice older than 30 weeks of age have renal dysfunction, making it difficult to survive TM treatment.

### Cell isolation and flow cytometry in mice spleen

Magnetic isolation of mouse splenic follicular B (FoB) cells, marginal zone B (MZ B) cells, and plasma cells was performed with the autoMACS Pro Separator (Miltenyi Biotec) using the Marginal Zone and Follicular B Cell Isolation Kit and the CD138^+^ Plasma Cell Isolation Kit (Miltenyi Biotec). Isolated cells were stained with Alexa Fluor 647-labeled (Molecular Probes, Eugene, OR, USA) MZB1 (Proteintech) and samples were analyzed using MACSQuant Analyzer (Miltenyi Biotec). For intracellular staining preparation, the PerFix-nc Kit (Beckman Coulter, Marseille, France) was used.

### Immunohistochemistry in mice

Mice organs were fixed in formalin and embedded in paraffin. Immunohistochemistry for MZB1 was performed and the number of MZB1^+^ cells was counted in organs including the submandibular gland, lung, liver, spleen, kidney, cecum, and intraperitoneal lymph node of aged and young BWF1 mice (*n* = 3 each) as already described for human samples.

### TM treatment

TM is an inhibitor of protein glycosylation, which blocks the initial step of glycoprotein biosynthesis in the endoplasmic reticulum (ER). TM treatment causes accumulation of unfolded glycoproteins that induces programmed cell death in response to ER stress [26]. BWF1 mice were given a single 1 μg/g body weight intraperitoneal injection of 0.05 mg/ml suspension of TM (Wako, Osaka, Japan) in 150 mM dextrose [27]. Mice spleens were subsequently removed at various time points post injection and subjected to immunoblotting for MZB1. BiP (C50B12; Cell Signaling, Danvers, MA, USA) and beta-actin were used as loading controls.

### Enzyme-linked immunosorbent assay for dsDNA in mice serum

The concentration of dsDNA in BWF1 mice (*n* = 7) serum taken serially during TM treatment was measured using the mouse anti-dsDNA ELISA KIT (Shibayagi, Gunma, Japan). B6 mice (*n* = 2) treated with TM were examined as controls.

### TUNEL (TdT-mediated dUTP nick end labeling) assay

Histochemical detection of fragmented DNA was performed using an in-situ apoptosis detection kit (Takara Bio, Shiga, Japan) in tissue sections of TM-treated BWF1 mice.

### Statistical analysis

Statistical analyses were performed using GraphPad Prism 6 (MDF Co., Ltd, Tokyo, Japan) or R version 3.2.0. (http://www.gnu.org/copyleft/gpl.html). The unpaired Student’s *t* test, the Mann–Whitney *U* test, or two-way analysis of variance (ANOVA) followed by Bonferroni correction were used. Data are presented as the means with standard error of the mean (SEM). *p* < 0.05 was considered statistically significant.

## Results

### MZB1 is one of the highly expressed proteins in SLE lymphadenopathy

**Fig. 1:**
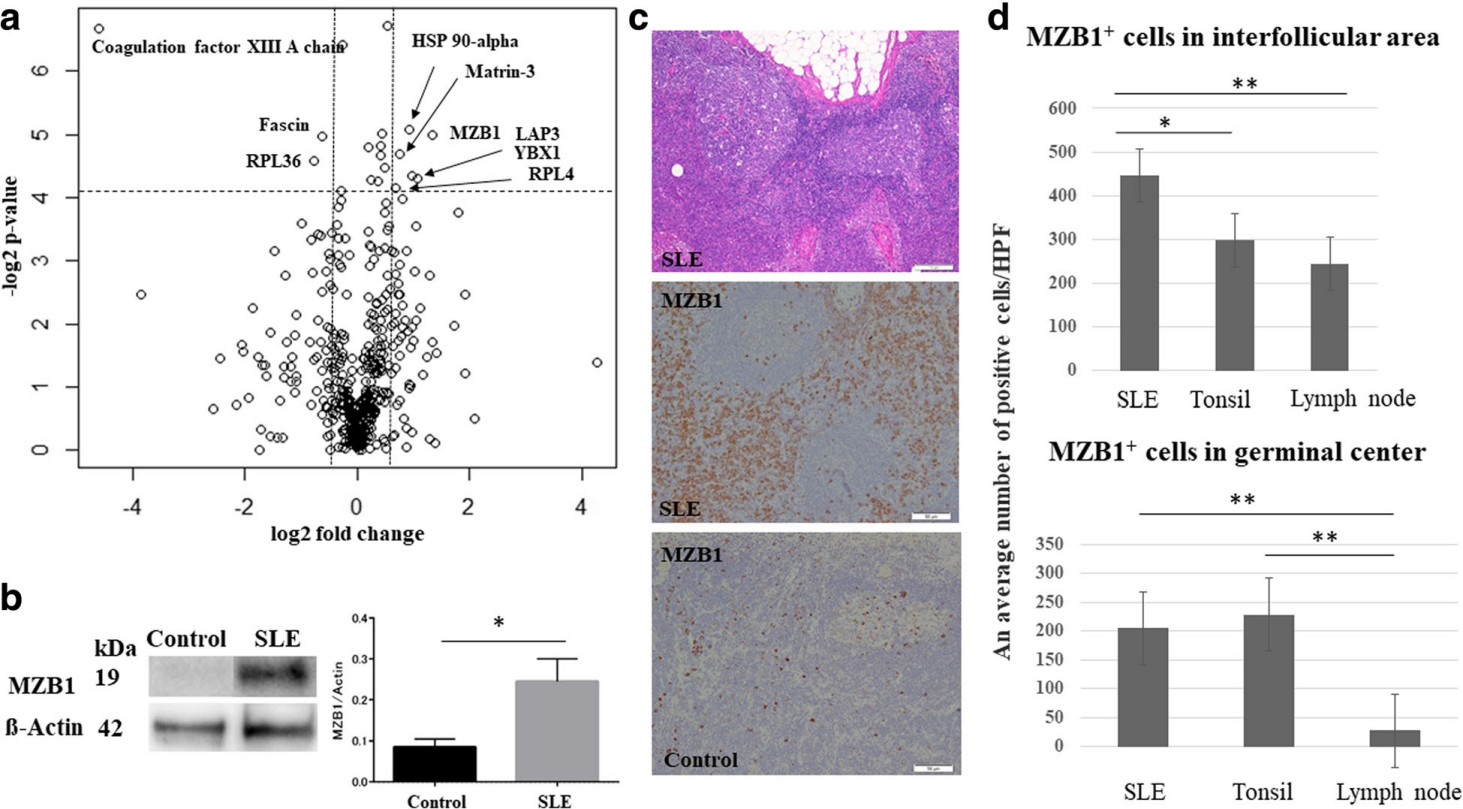
MZB1 is a highly expressed protein in lymph nodes from SLE patients. **a** Volcano plot showing distribution of all proteins in lymph nodes from SLE patients (*n* = 3) and controls (*n* = 3) analyzed by LC-MS. Horizontal line denotes fold change. Vertical line represents *p* value (ANOVA). Differences > 1.5-fold change and *p* ≤ 0.05 considered statistically significant. Fold-change values indicate higher (+) and lower (–) expression in SLE patients compared with controls. Significant proteins labeled with their gene name. **b** Representative immunoblotting for MZB1 in lymph node tissue from SLE patients and controls. Right: Quantification of the immunoblot (*n* = 3 each). Mean band intensity ratio measured as the intensity of the MZB1 band divided by intensity of the corresponding beta-actin band. Error bars indicate SEM. **c** Upper: histological section of an axillary lymph node from SLE patients showing reactive follicular hyperplasia. HE, scale bar = 100 μm. Middle: MZB1 immunostaining of the same sample of the upper image, showing numerous positive cells in the interfollicular area and within the germinal center. Scale bar = 50 μm. Lower: MZB1 immunostaining of the control. Scale bar = 50 μm. **d** MZB1^+^ cells in lymph nodes from SLE patients were significantly more frequently observed in the germinal center and interfollicular areas compared with those from controls. *p<0.05; **p<0.01 HPF high-power field, SLE systemic lupus erythematosus

LC-MS was performed on lymph nodes from SLE patients and controls (*n* = 3 each). Four hundred and sixty-five proteins were detected (Fig. 1a) (Additional file 1: Table S1). Highly confident (*p* ≤ 0.05) and differently expressed (>1.5-fold change) proteins between lymph nodes from SLE patients and controls were discovered in comparative proteomic studies. The intensity levels were increased in six proteins and decreased in three proteins in lymph nodes from SLE patients compared with controls (Table 1). One of the highly confident and differentially expressed proteins, MZB1, was chosen for a subsequent validation study because of its role in antibody secretion [28, 29].

**Table 1:** Candidate proteins differentially expressed between SLE patients and controls

UniProt Accession number	Protein name	Gene name	Location	Function	Fold change	ANOVA (*p*)
Q8WU39	Marginal zone B and B1 cell-specific protein	*MZB1*	ER	Immunoglobulin folding and secretion	2.5	0.03
P28838	Cytosol aminopeptidase	*LAP3*	Cytoplasm	Presumably involved in the processing and regular turnover of intracellular proteins	2.0	0.04
P07900	Heat shock protein HSP 90-alpha	*HSP90AA1*	Cytoplasm	Molecular chaperone	1.9	0.03
P43243	Matrin-3	*MATR3*	Nucleus	May play a role in transcription or may interact with other nuclear matrix proteins to form the internal fibrogranular network	1.7	0.03
P67809	Nuclease-sensitive element-binding protein 1	*YBX1*	Cytoplasm, nucleus, secreted	Extracellular mitogen and stimulates cell migration and proliferation	2.1	0.05
P36578	60S ribosomal protein L4	*RPL4*	Cytoplasm	Ribosomal protein	1.6	0.05
P00488	Coagulation factor XIII A chain	*F13A1*	Cytoplasm, secreted	Stabilizes fibrin clots	–24	0.01
Q9Y3U8	60S ribosomal protein L36	*RPL36*	Cytoplasm	Ribosomal protein	–1.7	0.04
Q16658	Fascin	*FSCN1*	Cytoplasm	Plays a role in the organization of actin filament bundles	–1.5	0.03

Proteins with > 1.5-fold change and *p* ≤ 0.05 (Mann–Whitney *U* test followed by the Bonferroni correction) were considered significant. Fold-change values indicate higher (+) or lower (–) expression in SLE patients compared with controls

UniProt/Swiss-Prot human proteomic database used as reference

*ANOVA* analysis of variance, *ER* endoplasmic reticulum

The validation study was performed using immunoblotting and immunohistochemistry for MZB1. This increased MZB1 expression in lymph nodes from SLE patients was confirmed by immunoblot analysis (Fig. 1b). A 3.1-fold increase in MZB1 expression levels was observed in specimens from SLE patients compared with those from controls (*p* < 0.05) (Fig. 1b). Representative images of hematoxylin and eosin (HE) staining and MZB1 immunohistochemistry in lymph nodes from SLE patients are shown in Fig. 1c. MZB1 was differentially expressed in lymph nodes from SLE patients compared with those from controls (*n* = 5 each) by immunohistochemistry. MZB1^+^ cells were observed in germinal centers and interfollicular areas in lymph nodes from SLE patients as well as controls (Fig. 1c). Moreover, the frequency of MZB1^+^ cells in lymph nodes from SLE patients was significantly higher both in germinal centers and interfollicular areas compared with those in control lymph nodes (*p* < 0.05) (Fig. 1d). MZB1^+^ cells in interfollicular areas were significantly increased in specimens from SLE patients compared with control tonsil specimens (*p* < 0.05) (Fig. 1d).

### MZB1 is expressed on plasma cells and MZ B cells

**Fig. 2:**
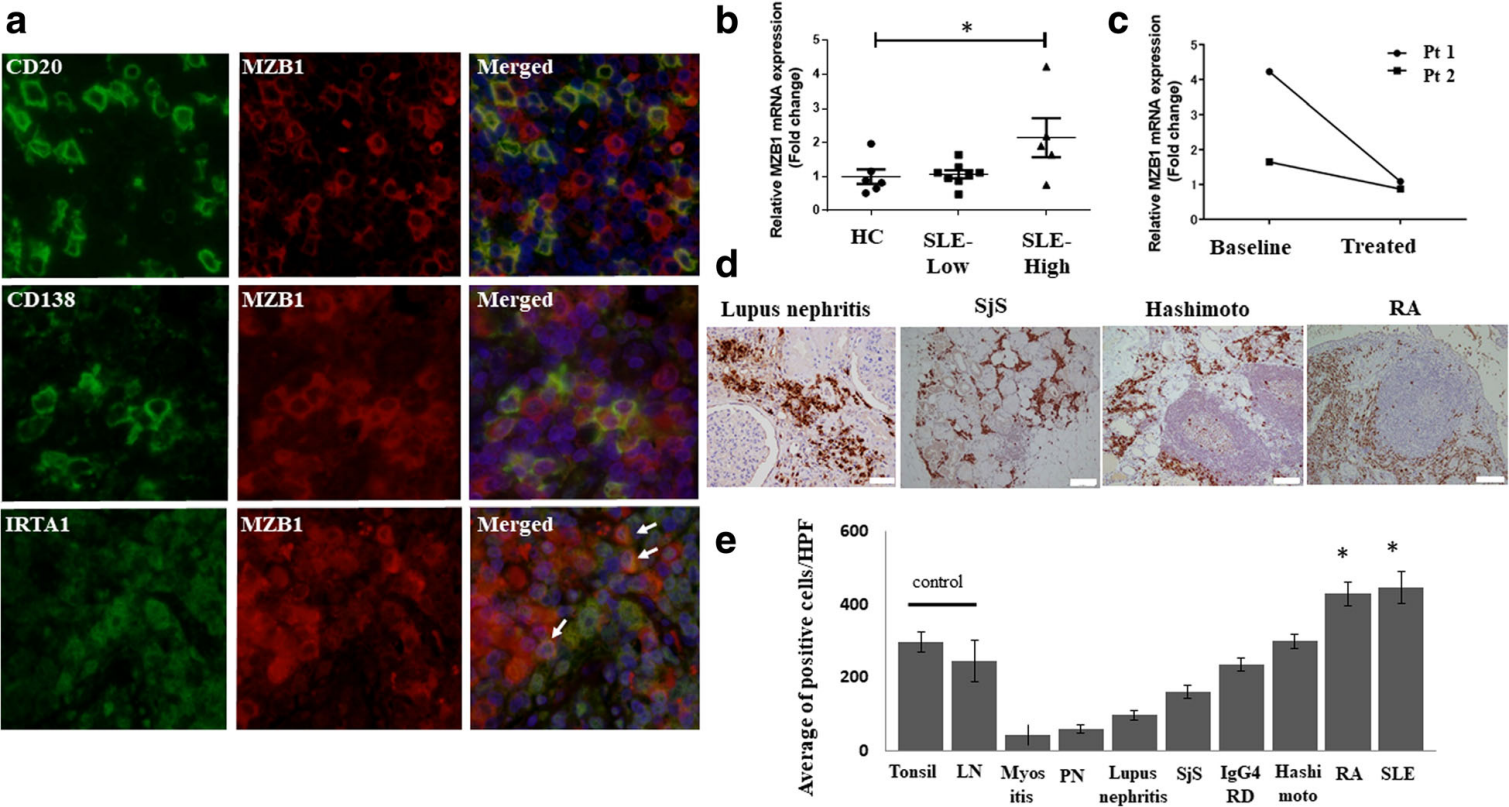
MZB1 is overexpressed in B-cell subsets and *MZB1* mRNA increased in peripheral blood B cells from SLE patients with active disease. **a** Immunofluorescence showed slight colocalization of MZB1 with B-cell marker CD20 and strong colocalization with plasma cell marker CD138 and MZ B-cell marker IRTA1 in lymph nodes from SLE patients. **b** *MZB1* mRNA levels in peripheral blood B cells from SLE patients with active disease (SLE-High) increased by 2.1-fold compared with those in healthy controls (HC) (*p* < 0.05). No significant increase in *MZB1* mRNA levels observed in peripheral blood B cells from SLE patients with inactive disease (SLE-Low). **c** Two SLE patients with active disease had follow-up samples collected at 2 months of treatment. Relative *MZB1* mRNA expression levels decreased with treatment. **d** MZB1 immunohistochemistry in tissue from patients with various autoimmune diseases. **e** Increased proportion of MZB1^+^ cells observed in lymph nodes from SLE patients and synovial tissue from rheumatoid arthritis (RA) patients compared with control lymph nodes (LN) and tonsils (*p* < 0.05). RA, scale bar = 50 μm; lupus nephritis, scale bar = 20 μm. Hashimoto Hashimoto’s thyroiditis, HPF high-power field, IgG4-RD IgG4-related pancreatitis, myositis dermatomyositis, PN polyarteritis nodosa, Pt patient, Sjs Sjögren’s syndrome, SLE systemic lupus erythematosus

Double immunostaining was performed on lymph node tissue from SLE patients to characterize MZB1-expressing cells. Significant colocalization of MZB1 and CD138 was observed, suggesting that MZB1 is predominantly expressed on plasma cells (Fig. 2a). Occasional MZB1 expression was observed on CD20^+^ B cells. IRTA1 [30], which is expressed on MZ B cells, colocalized with MZB1, suggesting that MZB1 is expressed on MZ B cells (Fig. 2a). IRTA1 is selectively expressed by a subpopulation of B cells in intraepithelial and subepithelial areas of human mucosa-associated lymphoid tissue, which express CD20^+^IgD^+^CD27^+^CD95^+^CD11b^+^CD5^–^CD23^–^CD38^–^ and carry mostly mutated but also unmutated IgV genes, which are regarded as human MZ B cells [30]. IRTA1 and CD138 expression is mutually exclusive, as MZ B cells differentiating into plasma cells generally lose IRTA1 expression [30].

### Increase in *MZB1* mRNA in peripheral blood B cells in SLE patients with active disease

Next, we examined *MZB1* mRNA expression of peripheral blood CD19^+^ B cells isolated from SLE patients and healthy donors. A 2.1-fold increase in *MZB1* mRNA expression was observed in peripheral blood B cells from SLE patients with active disease (SLEDAI-2 K ≥ 6) compared with those from healthy donors (*p* < 0.05). No increase in *MZB1* mRNA was observed in patients with inactive disease (SLEDAI-2 K < 6) (Fig. 2b). Two patients with active disease had follow-up samples collected at 2 months of treatment (Fig. 2c). One patient had SLEDAI-2 K scores of 14 at baseline and 4 at follow-up. The relative *MZB1* mRNA expression levels in this patient’s specimens decreased from 4.23 to 1.08, respectively. The other patient had SLEDAI-2 K scores of 10 at baseline and 0 at follow-up. The relative *MZB1* mRNA expression levels in this patient’s specimens decreased from 1.65 to 0.87, respectively.

### MZB1 immunohistochemistry for other autoimmune diseases

To examine whether MZB1 could discriminate SLE from other autoimmune diseases, MZB1 immunostaining in specimens from patients with other autoimmune diseases was performed (Fig. 2d). A significant increase in the proportion of MZB1^+^ cells was observed in lymph nodes from SLE patients and synovial tissue from rheumatoid arthritis patients compared with control lymph nodes and tonsils (*p* < 0.05) (Fig. 2e). There were no statistically significant increases in the number of MZB1-positive cells in patients with other autoimmune diseases.

### Splenic MZ B cells and plasma cells show elevated MZB1 levels in aged lupus-prone mice

**Fig. 3:**
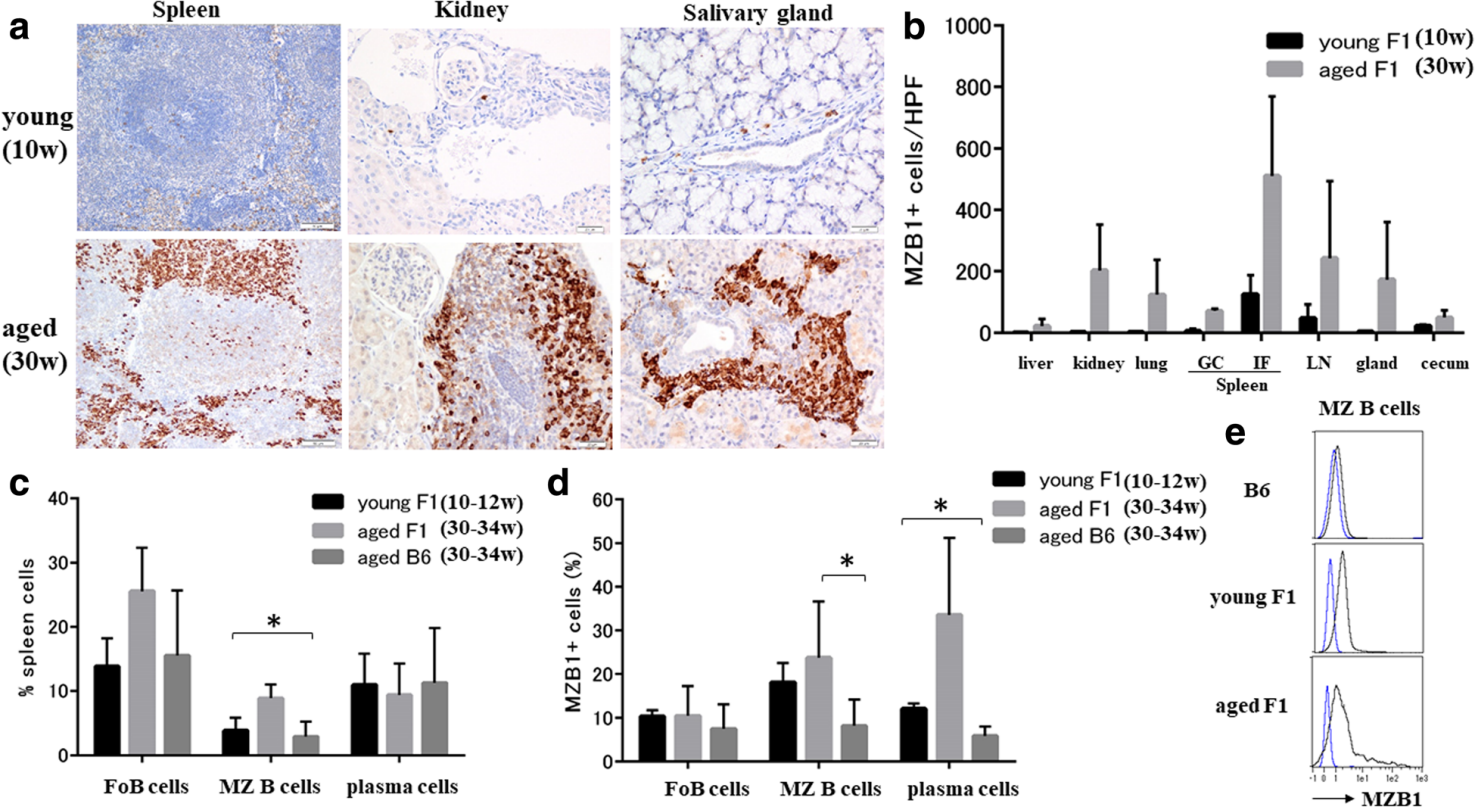
Splenic marginal zone B cells and plasma cells show elevated MZB1 levels in aged lupus-prone mice. **a** MZB1 immunohistochemistry in young (10 weeks of age) and aged (30 weeks of age) BWF1 mice. Spleen, scale bar = 50 μm; kidney, salivary gland, scale bars = 20 μm. **b** Increased proportion of MZB1^+^ cells observed in various organs in aged BWF1 mice compared with young BWF1 mice (ANOVA, *p* < 0.05). **c** Spleen cells sorted as B220^+^CD21^high^ CD23^low^ (MZ B cells) and B220^–^CD138^+^ (plasma cells). Percentages of B-cell subsets among total spleen cells in young (10–12 weeks of age) and aged (30–34 weeks of age) BWF1 mice compared with those in aged (30–34 weeks of age) B6 mice (*n* = 3–5 each group). Proportion of total MZ B cells increased on average by 8.8% in aged BWF1 mice compared with young BWF1 and B6 mice (ANOVA, *p* < 0.05). **d** MZB1 expression in each B-cell subset in young (10–12 weeks of age) and aged (30–34 weeks of age) BWF1 mice compared with aged (30–34 weeks of age) B6 mice (*n* = 3–5 each group). MZB1 expression in MZ B cells in aged BWF1 mice significantly higher than that in B6 mice (*p* < 0.05). In plasma cells, MZB1 expression in aged BWF1 mice significantly higher than that in young BWF1 and B6 mice (ANOVA, *p* < 0.05). **e** Representative histogram of MZB1 expression in MZ B cells in aged B6 mice and young and aged BWF1 mice. Blue line represents isotype control. GC germinal center, FoB follicular B, HPF high-power field, gland salivary gland, IF interfollicular area, LN lymph node, MZ B marginal zone B, w weeks, *p<0.05

Marked mononuclear cell infiltration in target organs including the liver, kidney, lung, submandibular gland, and submucosa of the cecum was observed in aged BWF1 mice. In immune organs (spleen and abdominal lymph nodes), expanded follicular and interfollicular areas were noted in aged BWF1 mice. A fewer inflammatory cells were observed in young BWF1 mice compared with aged BWF1 mice. An increased proportion of MZB1^+^ cells was observed in various organs in aged BWF1 mice compared with young BWF1 mice (ANOVA, *p* < 0.05) (Fig. 3a, b). In addition, MZB1^+^ cells were largely located inside the follicles in aged BWF1 mice compared with young BWF1 mice.

We next investigated the frequency of B-cell populations among total splenic cells in young and aged BWF1 mice compared with aged B6 mice (Fig. 3c). The proportion of total MZ B cells increased on average by 8.8% in aged BWF1 mice compared with young BWF1 and B6 mice (ANOVA, *p* < 0.05).

In addition, the proportion of MZB1^+^ MZ B cells in the spleen was significantly higher in aged BWF1 mice compared with those in aged B6 mice (*p* < 0.05) (Fig. 3d, e). In plasma cells, MZB1 expression in aged BWF1 mice was significantly higher than that of young BWF1 and B6 mice (ANOVA, *p* < 0.05) (Fig. 3d).

### ER stress with TM treatment induces apoptosis of MZB1^+^ cells in target organs resulting in decreased serum anti-dsDNA antibody levels

**Fig. 4:**
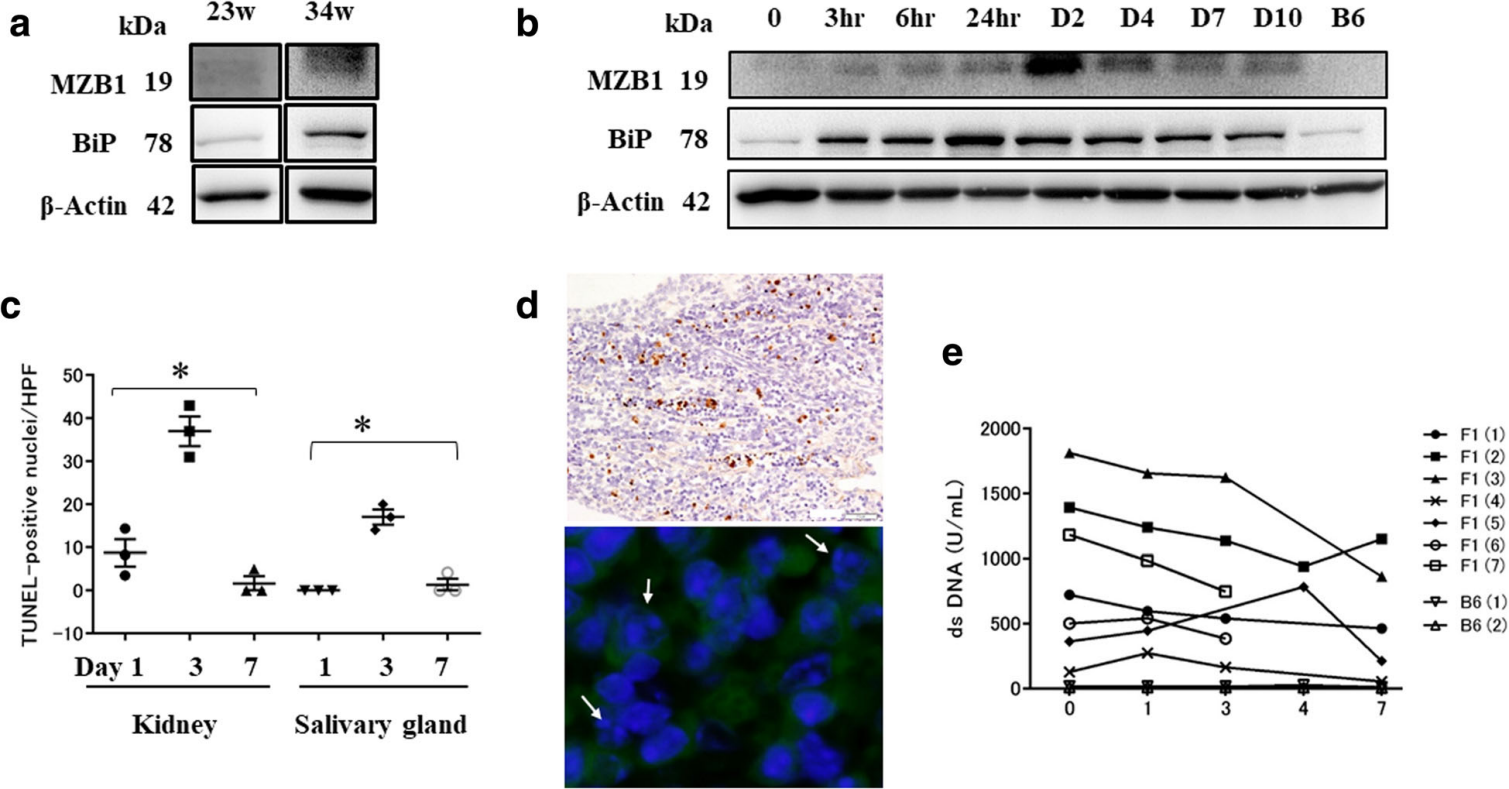
ER stress induces apoptosis of MZB1^+^ cells in target organs resulting in decreased serum dsDNA. **a** Baseline expression of MZB1 and BiP assessed on BWF1 mice at ages of 23 and 34 weeks and there were elevated levels at 34 weeks of age. **b** MZB1 and BiP expression in spleens from BWF1 mice (25 weeks of age) before or after indicated time points (hours and days) following TM treatment. Beta-actin used as a loading control. Representative blot of three independent experiments with similar results. **c** Number of TUNEL-positive cells per HPF in kidney and salivary gland of aged BWFI mice (30 weeks of age) serially taken after TM treatment was quantified. **d** (*Upper*) TUNEL-positive cells present among inflammatory cells in the kidney. (*Lower*) Condensed and fragmented nuclei in MZB1^+^ cells from BWF1 mice kidney following TM treatment. **e** Anti-dsDNA antibody concentrations in BWF1 mice (*n* = 7; 30 weeks of age) measured serially by ELISA following TM treatment. B6 mice (*n* = 2; 30 weeks of age) used as control. TUNEL, TdT-mediated dUTP nick end labeling; HPF, high-power field; hr, hour; *p<0.05

As MZB1 appears to associate with ER resident chaperones [28, 29, 31], aged BWF1 mice were treated with ER stress inducer TM 1 μg/g body weight for 7–10 days. BiP, which is highly expressed in the ER, was used as an ER marker [26]. Baseline MZB1 and BiP expression of BWF1 mice at the ages of 23 and 34 weeks is shown in Fig. 4a and there were elevated levels at 34 weeks of age. With TM treatment, BiP expression in the spleen peaked within 24 hours and continued to be upregulated until day 10. MZB1 expression peaked at 48 hours, followed by recovery on day 10 (Fig. 4b). Representative images of BWF1 mice at the age of 25 weeks are shown (Fig. 4b).

The unfolded protein response (UPR) signaling pathway is turned on when cells suffer from ER stress, but also when B cells differentiate into professional secretory cells (plasma cells) to facilitate proper protein folding [32]. When sustained or severe ER stress surpasses the capacity of UPR, the UPR promotes apoptosis [26]. Thus, we used the TUNEL assay to measure ER stress-mediated apoptosis in TM-treated aged BWF1 mice. TM treatment resulted in an increase in TUNEL-positive cells mostly within inflammatory cell aggregates in the interstitium, but not epithelial cells in various organs except renal proximal tubular epithelium [27]. The number of TUNEL-positive cells in renal medulla and salivary gland interstitial tissue peaked on day 3 and recovered by day 7 (Fig. 4c). Additionally, condensed and fragmented nuclei, indicating apoptotic cells, were observed in MZB1^+^ cells in TM-treated aged BWF1 mice (Fig. 4d).

To examine the effect of TM treatment, anti-dsDNA antibody levels were measured in aged BWF1 and B6 mice. In five of seven BWF1 mice, anti-dsDNA levels gradually decreased over the 7-day period after TM treatment. Two BWF1 mice died at day 3. One BWF1 mouse showed increased anti-dsDNA titers at day 4, which subsequently decreased by day 7. For control mice, there were no changes in anti-dsDNA concentrations during the treatment period (Fig. 4e).

## Discussion

MZB1, also known as pERp1, is an ER-associated protein that regulates B-cell receptor-driven calcium responses and is greatly induced during plasma cell differentiation. MZB1 regulates proper assembly and secretion of mature IgM. It is highly expressed in MZ B cells and B1 cells, with much lower expression in FoB cells in accordance with their role as prolific IgM secretors [28, 29, 31]. We found increased expression of MZB1 protein in SLE patient specimens as well as aged BWF1 mice specimens compared with respective controls, reflecting excessive autoantibody secretion in lupus. Our study confirmed MZB1 colocalized with CD138^+^ plasma cells and IRTA1^+^ MZ B cells in human tissues. In aged BWF1 mice, isolated splenic MZ B cells and plasma cells showed elevated levels of MZB1 compared with control mice. MZ B cells play important roles in the early phases of humoral immune responses. MZ B cells reside in follicles in lymphoid organs and efficiently transport blood-borne antigens to follicles, where they activate CD4^+^ T cells and rapidly differentiate into plasma cells [33–35]. In contrast, the majority of conventional B cells, termed FoB cells, produce specific antibodies with much slower kinetics. MZ B cells also contain a large number of autoreactive clones and the expansion of this compartment has been associated with autoimmunity [19]. The MZ B-cell compartment is expanded in BWF1 mice [16] and other murine models of lupus [15, 17, 18, 21]. Autoreactive MZ B cells enter follicles and interact with CD4^+^ T cells in lupus-prone mice [19, 20]. Increased intrafollicular localization of MZB1^+^ cells was observed in specimens from SLE patients as well as BWF1 mice compared with respective controls, suggesting the role of MZ B cells within follicles in lupus pathogenesis.

Prominent ectopic lymphoid tissue in target organs was observed in aged BWF1 mice, which was not seen in young BWF1 mice. Long-lived autoantibody-secreting cells are reportedly found within inflamed kidneys of BWF1 mice as well as SLE patients [36]. Excessive MZB1 expression in specimens from SLE patients and BWF1 mice may indicate MZB1 is a potential marker of prolific autoantibody-secreting B cells in autoimmune disease. Approximately 31% of renal IgG-producing cells react with dsDNA, which is higher than cells from the spleen (15%) and bone marrow (21%) in aged BWF1 mice [36]. Autoantibodies secreted by long-lived plasma cells in target organs were shown to be pathogenic and major drivers of inflammation in a study using adoptive transfer of antibody-secreting cells from BWF1 mice to Rag^–/–^ mice [37]. In our study, the frequency of MZB1^+^ cells in an isolated splenic B-cell subset was relatively lower than expected (at maximum, 34% of plasma cells) in aged BWF1 mice, while MZB1^+^ cells were predominantly observed in target organs, suggesting that survival niches for plasma cells exist in target organs [36].

The ER functions to properly fold and process secreted and transmembrane proteins. Physiological and pathological stimuli can disrupt ER homeostasis, resulting in an accumulation of misfolded and unfolded proteins, known as ER stress [26]. ER stress activates a complex signaling network referred as the UPR pathway to reduce ER stress, resulting in increased transcription of ER resident chaperones, folding enzymes, and components of the protein degradation machinery [26]. If misfolded proteins cannot be properly refolded, then they are sent outside the ER and subsequently degraded by cytosolic 26S proteasomes [32, 38]. BiP is a central regulator of UPR stress sensors as well as an ER chaperone that assists in protein folding. MZB1 is found to associate with ER resident chaperones and oxidoreductases, including BiP, GRP94, ERp57, calnexin/calreticulin, and PDIA6, and assists in folding of Igμ heavy chain [28, 29, 31]. Moreover, MZB1 appears to act as a cochaperone of GRP94 [39, 40]. MZB1 also promotes cell surface expression of integrins and Toll-like receptors, which are GRP94 clients [31, 39].

It is known that the UPR signaling pathway is turned on when B cells differentiate into plasma cells to facilitate proper protein folding [32]; however, under unresolvable ER stress conditions, the UPR pathway promotes apoptosis [26]. Our study showed BiP expression peaked within 24 hours, followed by recovery on day 10, in BWF1 mice after TM treatment. MZB1 expression peaked at 48 hours. BiP is ubiquitously expressed in cells and plays a role as a UPR stress sensor as well as an ER chaperone to assist protein folding, in contrast to MZB1 which is a B-cell-specific protein [26, 39, 40]. This may explain the earlier upregulation of BiP than MZB1. In TUNEL staining of tissue sections from TM-treated BWF1 mice, the number of TUNEL-positive cells peaked on day 3 and recovered by day 7. Serum anti-dsDNA antibody titers decreased with TM treatment, which may be due to apoptosis of B cells caused by excessive ER stress. One BWF1 mouse showed an increased anti-dsDNA antibody titer on day 4 compared with that before treatment; however, the titer decreased by day 7. ER stress may stimulate antibody-secreting cells to release antibodies soon after TM treatment; however, cell death may be induced under excessive ER stress, resulting in the decreased anti-dsDNA antibody titers observed on day 7. Apoptosis was predominantly observed in MZB1^+^ cells, suggesting that TM treatment mainly affected antibody-secreting cells.

Because chaperones can associate with a number of proteins, including autoantigens, they can act as endogenous adjuvants in immune responses [41]. ER resident chaperones BiP, GRP94, calnexin, and HSP90 take part in MHC peptide loading in addition to their roles in folding and assembly of early intermediates of MHC class I and II molecules, and thus they may be involved in antigen recognition and presentation [42]. Some ER resident chaperones, such as GRP94, are released into extracellular fluid, likely by destruction of the plasma membrane that accompanies stress-induced apoptosis or necrosis [41]. Autoantibodies against BiP, GRP94, calnexin, and HSP90 are detected in SLE and RA patients [43–45], although their pathophysiological role remains unknown. In this study, we determined serum anti-MZB1 antibody concentrations by ELISA and found no significant difference between SLE patients and healthy donors (data not shown). This may be because MZB1 is expressed at a lower concentration than ubiquitously expressed BiP, GRP94, and HSP90 [26].

The observations described in this study implicate an endogenous chaperone in the pathogenesis of SLE, and the selective nature and excessive expression of MZB1 in B-cell subsets make it an attractive therapeutic target for SLE treatment [46]. Knockdown of MZB1 slows down IgM polymerization and subsequently leads to reduced IgM secretion [29, 31]. Recently, HSP90 inhibitors were suggested to be effective against a variety of oncogene-addicted cancers [47]. Bortezomib treatment has been demonstrated to be effective in refractory SLE patients [11]. Bortezomib is a selective inhibitor of the 26S proteasome, which is present in all eukaryotic cells, and thereby it exhibits systemic toxicity [11, 48]. In contrast, MZB1 is limitedly expressed in MZ B cells, B1 cells, and plasma cells. MZB1 inhibitors may cause degradation of immunoglobulin, causing apoptosis of autoantibody-secreting cells in SLE and other autoimmune diseases.

## Conclusions

MZB1, an ER resident molecular chaperone, was overexpressed in B-cell subsets in lymph nodes from SLE patients and lupus-prone mice. MZB1 may be a potential therapeutic target in excessive antibody-secreting cells in SLE.

## Additional file

Additional file 1: Table S1. Proteomic information of 465 proteins detected by LC-MS. (XLSX 127 kb)

## Abbreviations

ANOVA: Two-way analysis of variance
B6: C57BL/6 N
BWF1: [NZB × NZW] F1
ELISA: Enzyme-linked immunosorbent assay
ER: Endoplasmic reticulum
FFPE: Formalin-fixed paraffin-embedded
FoB: Follicular B
HPF: High-power field
LC-MS: Liquid chromatography-tandem mass spectrometry
MZ B: Marginal zone B
qRT-PCR: Quantitative real-time PCR
SEM: Standard error of the mean
SLE: Systemic lupus erythematosus
SLEDAI-2 K: SLE Disease Activity Index 2000
TM: Tunicamycin
TUNEL: TdT-mediated dUTP nick end labeling
UPR: Unfolded protein response
